# Binding of the feature *stimulus duration* in the auditory domain: S-R or S-S binding; or both?

**DOI:** 10.1177/17470218241287190

**Published:** 2024-10-23

**Authors:** Katrin Köllnberger, Johanna Bogon, Gesine Dreisbach

**Affiliations:** 1 Department of Psychology, University of Regensburg, Regensburg, Germany; 2 Media Informatics Group, University of Regensburg, Regensburg, Germany

**Keywords:** Feature binding, partial repetition costs, temporal features, stimulus-stimulus binding, stimulus-response binding, time perception

## Abstract

Perceiving and reacting to multidimensional objects creates so-called event files via feature binding. Bogon, Thomaschke, and Dreisbach provided the first evidence for the integration of the feature *stimulus duration* into such event files. However, their paradigm did not allow for differentiation between stimulus-stimulus and stimulus-response binding. This study used a classification task with many-to-one mappings to examine the integration of stimulus and response features independently. Experiment 1 used a pitch classification task. Participants had to respond with a left keypress to a low and a very low sine tone and with a right keypress to a high and very high sine tone. The four sine tones were presented for either a short or long duration, resulting in a total of eight stimuli. As an indicator of binding, we used partial repetition costs (better performance when both pitch/response and duration repeat or shift relative to partial repetitions). Results of Experiment 1 indicate stimulus-response binding and no stimulus-stimulus binding. In Experiment 2, instead of classifying the pitch of artificial sine tones, participants had to classify the type of musical instruments that produced the stimulus tones. Results replicated evidence for stimulus-response binding but also provided indications for stimulus-stimulus binding. Potential reasons for this result pattern and the relevance of duration in a musical context as one potential moderator of stimulus-stimulus bindings are discussed.

To perceive and interact with our environment appropriately, we need to temporally bind the different features of an object/event to form a coherent representation. Such binding processes have already been demonstrated for various modalities (visual, auditory, and multimodal) and different feature combinations, including stimulus-stimulus (S-S) bindings, stimulus-response (S-R) bindings, and response-response (R-R) bindings (for reviews, see Frings et al., 2020; Spence & Frings, 2020). Although most studies have demonstrated the binding of static features, recent evidence suggests that dynamic features, such as duration, are also bound (Bogon et al., 2023; Bogon, Thomaschke, & Dreisbach, 2017; Köllnberger et al., 2023; Mocke et al., 2022; Pfister et al., 2022). However, it is still unknown whether duration as a stimulus feature is bound to another stimulus feature, to the response feature, or possibly to both. The following study aims to clarify this question.

## Object perception and the binding problem

When we hear a smoke alarm beeping, this event consists of several different features, such as the pitch, volume, melody, and duration of the sound; its location (e.g., from the kitchen); and possible appropriate responses, such as evacuating the dwelling and calling the fire department. In our everyday life, we are able to perceive objects and events of different modalities as a coherent unit and, if necessary, react to them, although the features that form such objects and events are usually processed in different parts of the brain (Felleman & van Essen, 1991; Jeannerod, 1999; Mesulam, 1998). The binding problem addresses the question of how these different features become a temporary coherent representation (Treisman, 1996, 1998, 1999). Some evidence suggests that the various perceptual and action features of an event are temporarily bound by episodic bindings (Henson et al., 2014; Hommel, 1998, 2004; Kahneman et al., 1992; Treisman, 1996, 1999). Treisman refers to this temporary binding of perceptual features as an “object file,” a temporary episodic representation of the object that contains the traces of the distributed feature representations (*feature integration theory*, Kahneman et al., 1992; Treisman, 1996, 1998, 1999; Treisman & Gelade, 1980). An extension of these object files is so-called “event files,” which go beyond the binding of perceptual features and also include bindings of response-related features, as first formulated in the theory of event coding (Hommel, 2009; Hommel et al., 2001).

A commonly used measure to identify bindings between stimulus features (S-S binding) or between stimulus features and response features (S-R binding) is *partial repetition costs*; participants respond slower and more error-prone to a current event when only some of the features of the previous event are repeated/shifted (partial repetition) than when all features are repeated or shifted (complete repetition/shift). A partial repetition of the features retrieves the previous binding of the features and interferes with the setup of the current features of the event. The previous binding must first be detached to form a new binding, resulting in longer reaction times (RTs) and more errors. When all the features are repeated, the previous binding can simply be retrieved and responded to adequately. In the case of a complete shift of features, no interference with the previous event/binding occurs (e.g., Bogon, Eisenbarth, et al., 2017; Bogon, Thomaschke, & Dreisbach, 2017; Frings, C., Moeller, B., & Rothermund, K. (2013)’ and ‘Frings, C., Rothermund, K., & Wentura, D. (2007) Giesen & Rothermund, 2014; Henson et al., 2014; Hommel, 1998, 2004; Kahneman et al., 1992; Mayr et al., 2011; van Dam & Hommel, 2010; Zmigrod & Hommel, 2009, 2010).

## S-S and S-R binding

Bindings between stimulus features have already been demonstrated for a variety of features. Partial repetition costs have been found for visual stimulus features such as shape and colour, word identity, letter and picture identity, and facial features, as well as for real and abstract objects (e.g., Colzato et al., 2006; Hommel, 1998; Kahneman et al., 1992; Keizer et al., 2008; Tipper & Cranston, 1985; Waszak et al., 2003). Partial repetition costs have also been shown for auditory features such as pitch and loudness, vocal features, and sound identity, as well as for spatial features of visual and auditory stimuli (e.g., Herwig & Waszak, 2012; Hommel, 1998; Maybery et al., 2009; Zmigrod & Hommel, 2009). Furthermore, binding effects are found for both task-relevant and task-irrelevant stimulus features (e.g., Hommel, 1998; Horner & Henson, 2011; Mayr et al., 2009; Moeller et al., 2012; Waszak et al., 2003).

In addition to perceptual features, an event usually also consists of response-related features. Therefore, bindings can occur not only between stimulus features (S-S binding) but also between a stimulus and a response (S-R binding). Such S-R bindings have been demonstrated repeatedly over the years (see Spence & Frings, 2020, for a review). S-R bindings have been demonstrated for response locations, response features such as effector identity, valences of actions, and voice features, among others (e.g., Bogon, Eisenbarth, et al., 2017; Eder & Klauer, 2007; Hommel, 1998; Stoet & Hommel, 1999).^1^

## Integration of the feature duration

Besides the impressive number of features for which binding processes have already been shown (see previous sections), it is striking that the feature duration has barely been studied so far. This may be due to the fact that duration, unlike most features, is dynamic. The duration of a stimulus can only be fully defined when the presentation of the stimulus ends, i.e., the feature duration constantly re-updates itself. In contrast, a static feature, e.g., colour or pitch, can be defined upon its first appearance. Another characteristic that distinguishes time from other features is its anisotropy, which means that the direction of perceived time cannot be manipulated. We can change the dimension of features, such as sounds, continuously in both directions, i.e., we can turn a sound louder and softer, but we cannot influence the dimensional direction of the perceived flow of time (Riemer, 2015; Riemer et al., 2012).

In recent years, the feature *duration* has attracted increasing attention. After providing the first evidence of the integration of stimulus duration into auditory event files (Bogon, Thomaschke, & Dreisbach, 2017), binding processes have also been shown for temporal response features (Bogon et al., 2023; Mocke et al., 2022; Pfister et al., 2022), temporal expectancy (Schmalbrock & Frings, 2022), and the integration of stimulus duration into visual event files (Köllnberger et al., 2023).

Bogon, Thomaschke, and Dreisbach (2017) showed that stimulus duration is temporarily integrated into auditory event files. More specifically, they investigated the binding of stimulus duration to pitch (Experiment 1) and to loudness (Experiment 2). For this purpose, they used classification tasks with one-to-one mappings; participants had to respond to a high-pitch tone (loud tone) with a right keypress and to a low-pitch tone (soft tone) with a left keypress. The stimuli could appear either for a short or longer duration. Both experiments showed partial repetition costs, indicating the integration of stimulus duration into auditory event files. However, and relevant to the study presented here, due to the one-to-one mappings in the classification tasks used by Bogon, Thomaschke, and Dreisbach (2017), every stimulus shift (pitch or loudness) was also a response shift, and every stimulus repetition (pitch or loudness) was also a response repetition. Therefore, one cannot distinguish whether *duration* was bound to the other stimulus feature (*pitch, loudness*), to the *response*, or to both.

## Present study

The purpose of this study is to fill this gap and investigate whether stimulus duration in auditory event files is bound to another stimulus feature (S-S binding), to the response (S-R binding), or to both. To consider the stimulus features and the response features independently of each other, we used a many-to-one mapping design (see Giesen & Rothermund, 2011; Moeller et al., 2016; Moeller & Frings, 2014); two stimuli were assigned to each of two response keys. In Experiment 1, participants completed a sine tone classification task by responding to the two different low tones with the left response key and to the two different high tones with the right response key. All four stimuli (sine tones) could be presented either for a short (50 ms) or long duration (200 ms; cf. Bogon, Thomaschke, & Dreisbach, 2017). The feature *pitch* was task-relevant, and the feature *duration* was task-irrelevant. If *duration* is bound to *pitch* (S-S binding), sequential analysis of error rates and reaction times should reveal a partial repetition costs pattern. Repetition of *duration* paired with repetition of *pitch* from trial *n* − 1 to trial *n* should result in better performance than repetition of *duration* paired with *pitch* shift. Similarly, a complete shift of stimuli should lead to better performance than a partial repetition/shift of features. If the *duration* is bound to the *response* (S-R binding), an equivalent pattern should emerge for the *response*. If the *duration* is bound to both the *pitch* and the *response*, partial repetition costs patterns should emerge for each. The second experiment is a conceptual replication of the first experiment. In Experiment 2, participants responded to more qualitative auditory stimuli, namely sounds of musical instruments.

## Experiment 1

### Material and methods

#### Participants

An a priori power analysis (MorePower 6.0.4, Campbell & Thompson, 2012) revealed that to detect a two-way interaction effect size of $\eta_{p}^{2}$ = .42 (the minimum interaction effect size from Bogon, Thomaschke, & Dreisbach, 2017) with a power of 1 − β = .95, α = .05, and mean squared error﻿ (MSE) = 1, a minimum sample size of *N* = 20 would be necessary. To ensure that we would obtain the minimum number of required participants even after potential exclusions, we aimed for a minimum number of 30 participants for each experiment. We used the effect size from Bogon, Thomaschke, and Dreisbach (2017) because their study investigated the binding of duration in an auditory context, which is very close to the topic of the present study. Our sample size of 30 participants was also sufficient to detect smaller effect sizes of $\eta_{p}^{2}$ ⩾ .23 with a power of 1 − β = .80. It is worth noting, although, that an effect size of $\eta_{p}^{2}$ ⩾ .14 is considered large (Cohen, 1988), so an effect size of $\eta_{p}^{2}$ ⩾ .23 still represents a relatively large effect.

Thirty students (age *M* = 22.0 years, *SD* = 5.18; range = 18–39 years; 24 self-identified as female, six as male; one left-handed [self-report]) from the University of Regensburg participated for course credit. They all gave written informed consent prior to the experiment in accordance with the ethical standards of the National Research Committee and with the 1964 Helsinki Declaration and its later amendments. All participants reported no hearing impairment.

#### Apparatus and stimuli

The experiment was run in E-Prime (Version 2.0, Psychology Software Tools, Sharpsburg, PA, USA). The stimuli consisted of four pure sine tones (200 Hz, 400 Hz, 800 Hz, and 1,000 Hz) with two durations (50 ms and 200 ms), resulting in a total of eight stimuli. Instructions and stimuli were presented on a 21.5-inch monitor (Dell Inc., Round Rock, TX, USA). The sine tones were played via headphones (HD 201 Sennheiser, Wedemark, Germany) at a constant volume of 78 dB throughout the experiment. Participants were instructed to respond to the “very low” and “low” tones using the left response key and to the “high” and “very high” tones using the right response key (“Y” and “M” keys on a standard QWERTZ keyboard, many-to-one mapping), positioned centrally in front of the participant. This fixed assignment was chosen based on the results of Rusconi et al. (2006) because low tones tend to be associated with the left, and high tones tend to be associated with the right. The fixation cross was a plus sign (black, 28 pt, Courier New, bold), and feedback was only given for errors (“Fehler” in red, 18 pt, Arial, bold). All stimuli were presented on a grey background (RGB: 192, 192, 192).

#### Procedure

**Figure 1.**
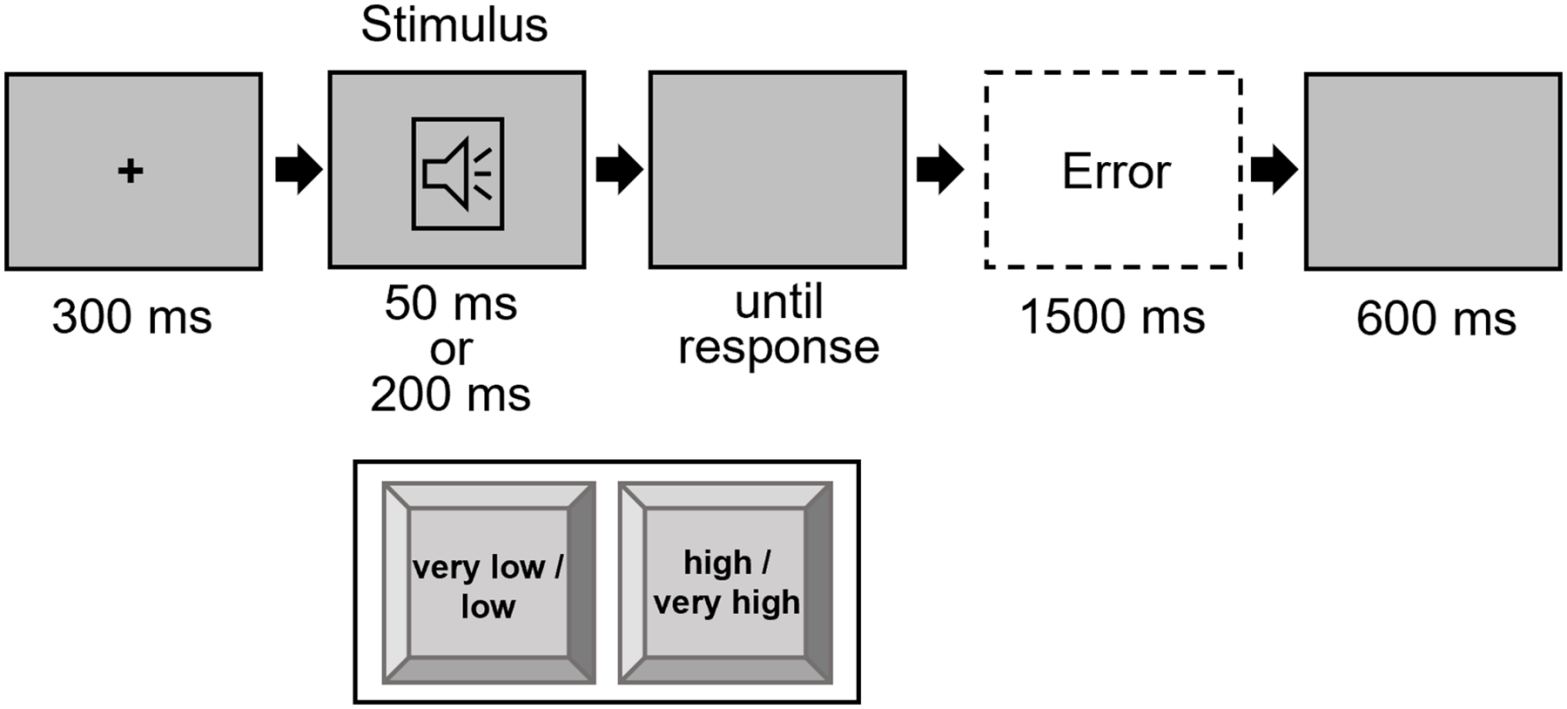
Trial procedure in Experiment 1. *Note.* The loudspeaker symbol is only for visualisation and was not a visual part of the trial. Stimuli are not drawn to scale.

Each trial started with a fixation of 300 ms duration. Then, the target stimulus was presented either for 50 ms or 200 ms, accompanied by a blank screen that was visible until the response was given. The participants were able to give a response from the beginning of the target. After an inter-trial interval of 600 ms, the next trial started. If the answer was incorrect, an error message appeared for 1,500 ms (see Figure 1). The experiment consisted of two practice blocks: the first with 20 trials and the second with 40 trials, followed by five experimental blocks of 128 trials each. The trial order was randomised, with the constraint that in the experimental blocks, each possible factor combination^2^ (*pitch* sequence × *duration* sequence × *response* sequence) appeared at least 14 times. In the first practice block, the participants were introduced to one of the high and low tones by hearing them once. Subsequently, the participants could practise the task (low-left; high-right) across 20 trials. In the second practice block, the remaining two tones (very low and very high) were introduced, followed by 40 trials, allowing practice with all four stimuli before proceeding to the experimental blocks. Participants were instructed to respond as fast and accurately as possible. Between blocks, there were breaks of 30 s.

### Design and planned statistical analyses

Our design included three within-subject factors, each with two levels: Pitch (repetition vs. shift), Duration (repetition vs. shift), and Response (repetition vs. shift). Any shift in pitch, whether within a response category or across response categories, was coded as a pitch shift (e.g., very low—low = shift and very low—high = shift). As a repetition of the response-relevant feature necessarily had to be answered with the same response (response repetition), the combination “pitch repetition and response shift” was not present (see Table 1 for an overview of possible condition combinations). Therefore, instead of an overall three-factorial design, we used separate two-factorial designs to investigate the respective Feature × Feature and Feature × Response interactions. For investigating S-S binding, we conducted a 2 (*Pitch*: repetition vs. shift) × 2 (*Duration*: repetition vs. shift) analysis of variance (ANOVA) with repeated measures on both factors for *response repetition trials only*. If duration is bound to another stimulus feature, we should find a Duration × Pitch interaction and thus partial repetition costs. For investigating S-R binding, we conducted a 2 (*Response*: repetition vs. shift) × 2 (*Duration*: repetition vs. shift) ANOVA with repeated measures on both factors for *pitch shift trials only.* If duration is bound to the response, we should find a Duration × Response interaction and thus partial repetition costs. The interactions of interest are further augmented by the Bayesian factor BF_incl_ (using the “matched models” method suggested by Sebastiaan Mathôt; JASP Team, 2022).

**Table 1.** Possible condition combinations in Experiments 1 and 2.

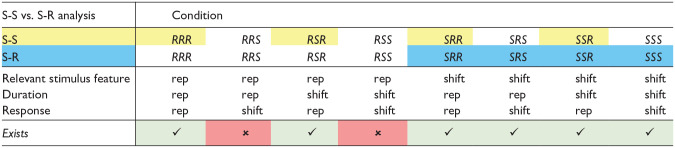

*Note.* Conditions marked with a “checkmark” exist, whereas conditions marked with an “X” do not exist. The relevant stimulus feature in Experiment 1 is pitch, and in Experiment 2, it is instrument. All data points from condition combinations marked in yellow are included in the S-S analysis, and all data points from condition combinations marked in blue are included in the S-R analysis. “rep” indicates repetition.

Furthermore, we calculated the *Binding Effect* for S-S and S-R binding by subtracting the potential interference from the potential benefit. More precisely, the S-S binding effect is composed as follows: [PRDS—PRDR]—[PSDS—PSDR]^3^. The same procedure applies to the S-R binding effect: [RRDS—RRDR]—[RSDS—RSDR]^4^ (e.g., Moeller et al., 2019; Moeller & Frings, 2019b; Pfister et al., 2019). Binding effects were compared using post hoc one-sample *t*-tests (two-sided), supplemented by Bayesian *t*-tests with the Bayes factor BF_10_, which quantifies the evidence for the alternative hypothesis in relative terms to the evidence for the null hypothesis.

All aforementioned analyses were planned and determined before the experiment, i.e., a priori. Raw data files associated with this article can be found online (https://doi.org/10.5283/epub.58056).

### Results and discussion

We analysed data from the five experimental blocks, excluding the first trial of each block from the analysis. Error trials (6.71%), trials following an error trial (7.39%), trials with extreme RTs < 100 ms or >8000 ms^5^ (0.01%), and trials with RTs deviating more than three *SDs* from the individual condition mean (1.63%) were excluded from the reaction time (RT) analysis (Bush et al., 1993). Data were analysed using SPSS statistical software (IBM Corp, 2017). Bayesian analyses were conducted with the program JASP (JASP Team, 2022). Bayes factors were categorised following the approach of Wagenmakers et al. (2011) and Wetzels et al. (2011).

#### S-S binding

##### RT data

**Figure 2.**
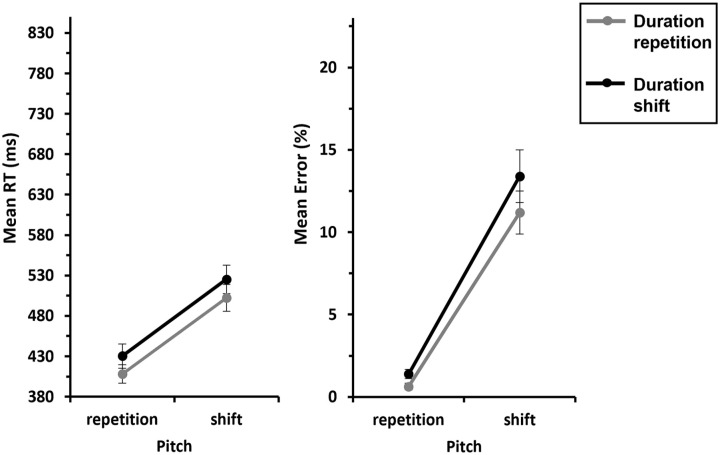
Mean RTs and errors of Experiment 1 as a function of Pitch x Duration—S-S binding. *Note.* Mean RTs (left panel) and mean error rates (right panel) as a function of Pitch (repetition vs. shift) and Duration (repetition vs. shift). Note that there are no response shifts for pitch repetitions, so we only included response repetitions in this analysis. The grey lines represent duration repetitions, and the black lines represent duration shifts. Error bars provide standard errors.

We conducted a 2 (*Pitch*: repetition vs. shift) × 2 (*Duration*: repetition vs. shift) ANOVA with repeated measures on both factors, for *response repetition trials*. This revealed a significant main effect of Pitch, *F*(1, 29) = 136, *p* < .001, $\eta_{p}^{2}$ = .824, indicating faster responses when pitch was repeated compared with when pitch shifted (419 ms vs. 514 ms). The main effect of Duration also reached significance, *F*(1, 29) = 26.6, *p* < .001, $\eta_{p}^{2}$ = .479, indicating faster responses when duration was repeated compared with when duration shifted (455 ms vs. 478 ms). However, the interaction of interest, Pitch × Duration, was not significant (*F* = 0.012, *p* = .914, $\eta_{p}^{2}$ < .001; see Figure 2, left panel). The inclusion Bayes factor (BF_incl_ = 0.285) for this interaction suggests that the data are 0.285 times more likely under the alternative hypothesis (interaction) than under the null hypothesis (no interaction), indicating substantial evidence for the H_0_.

##### Error rates

An analogous ANOVA for errors yielded a significant main effect of Pitch, *F*(1, 29) = 73.1, *p* < .001, $\eta_{p}^{2}$ = .716, indicating that overall, participants made more errors when pitch shifted compared with when it repeated (12.3% vs. 1.01%). Likewise, the main effect for Duration was also significant, *F*(1, 29) = 8.24, *p* = .008, $\eta_{p}^{2}$ = .221, indicating more errors for duration shifts compared with duration repetitions (7.39% vs. 5.91%). However, the interaction of interest, Pitch × Duration, reached no significance (*F* = 2.22, *p* = .147, $\eta_{p}^{2}$ = .071; see Figure 2, right panel). The inclusion Bayes factor (BF_incl_ = 0.367) for this interaction suggests that the data are 0.367 times more likely under the alternative hypothesis (interaction) than under the null hypothesis (no interaction), indicating anecdotal evidence for the H_0_.

#### S-R binding

##### RT data

**Figure 3.**
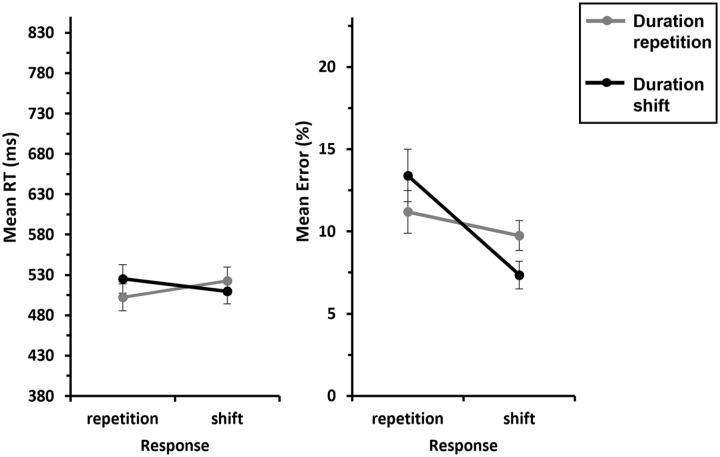
Mean RTs and errors of Experiment 1 as a function of Response × Duration—S-R binding. *Note.* Mean RTs (left panel) and mean error rates (right panel) as a function of Response (repetition vs. shift) and Duration (repetition vs. shift). Note that there are no pitch repetitions for response shifts, so we only included pitch shifts in this analysis. The grey lines represent duration repetitions, and the black lines represent duration shifts. Error bars provide standard errors.

We conducted a 2 (*Response*: repetition vs. shift) × 2 (*Duration*: repetition vs. shift) ANOVA with repeated measures on both factors for *pitch shift trials*. This revealed a significant Response × Duration interaction, *F*(1, 29) = 43.8, *p* < .001, $\eta_{p}^{2}$ = .601, indicating partial repetition costs: responses were faster when the response and duration were repeated compared with when the response repeated and duration shifted (502 ms vs. 525 ms). Conversely, a complete shift of response and duration led to faster responses compared with a response shift and a duration repetition (510 ms vs. 523 ms; see Figure 3, left panel). The inclusion Bayes factor (BF_incl_ = 10.7) for this interaction confirms this, indicating that the data are 10.7 times more likely under the alternative hypothesis (interaction) than under the null hypothesis (no interaction), suggesting strong evidence for the H_1_. None of the main effects were significant (all *F*s < 2.20 and *p*s > .149).

##### Error rates

An analogous ANOVA for errors yielded a significant main effect of Response, *F*(1, 29) = 7.66, *p* = .010, $\eta_{p}^{2}$ = .209, indicating that overall, participants made more errors when the response repeated compared with when it shifted (12.3% vs. 8.55%). The interaction of interest, Response × Duration, also reached significance, *F*(1, 29) = 20.9, *p* < .001, $\eta_{p}^{2}$ = .419, indicating partial repetition costs: responses were less error-prone when the response and duration were repeated compared with when response repeated and duration shifted (11.2% vs. 13.4%). Conversely, a complete shift of response and duration resulted in fewer errors compared with shifting the response and repeating the duration (7.35% vs. 9.74%; see Figure 3, right panel). The inclusion Bayes factor (BF_incl_ = 3.50) for this interaction confirms this, indicating that the data are 3.50 times more likely under the alternative hypothesis (interaction) than under the null hypothesis (no interaction), suggesting substantial evidence for the H_1_. The main effect of Duration was not significant (*F* = 0.020, *p* = .888, $\eta_{p}^{2}$ = .001).

#### Binding effect

**Figure 4.**
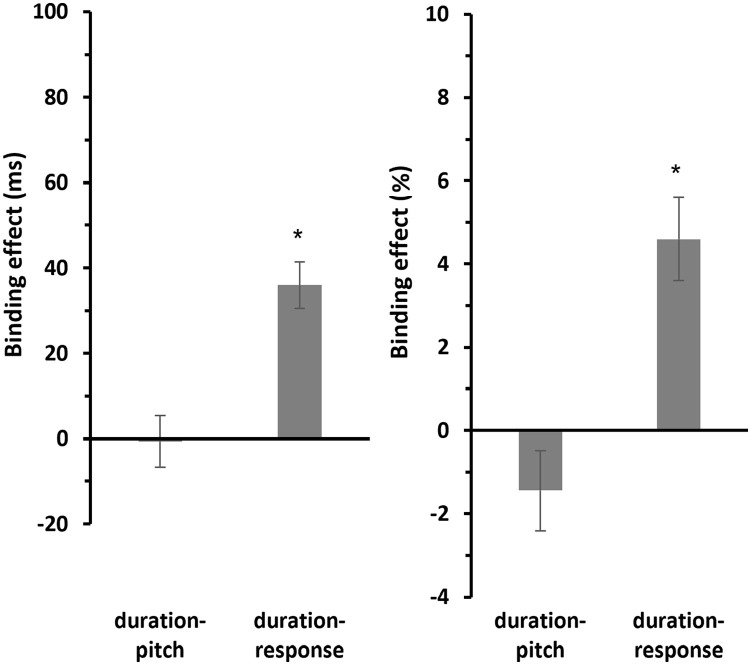
Mean binding effects for RTs and errors of Experiment 1. *Note.* Mean binding effects for RTs (left panel) and errors (right panel). For calculation of the binding effect, see text. Error bars provide standard errors.

Post hoc one-sample *t*-tests (two-sided) revealed that the binding effect for duration-pitch binding (*M* = −0.655 ms, *SE* = 6.03 ms) was not significantly different from zero, *t*(29) = −0.108, *p* = .914, *d* = −0.02, 95% confidence interval [CI] for Cohen’s *d* [−0.378, 0.338], and BF_10_ = 0.195. In contrast, the binding effect for duration-response binding (*M* = 35.9 ms, *SE* = 5.43 ms) was significant, *t*(29) = 6.62, *p* < .001, *d* = 1.21, 95% CI for Cohen’s *d* [0.729, 1.68], and BF_10_ = 54,005 (see Figure 4, left panel).

A similar pattern has been observed for the error rates (see Figure 4, right panel). Post hoc one-sample *t*-tests (two-sided) showed that the binding effect for duration-pitch binding (*M* = −1.45%, *SE* = 0.969%) was not significantly different from zero, *t*(29) = −1.49, *p* = .147, *d* = −0.272, 95% CI for Cohen’s *d* [−0.635, 0.095], and BF_10_ = 0.526. In contrast, the binding effect for duration-response binding (*M* = 4.60%, *SE* = 1.01%) was significant, *t*(29) = 4.57, *p* < .001, *d* = 0.834, 95% CI for Cohen’s *d* [0.412, 1.246], and BF_10_ = 305.

The results of Experiment 1 suggest that the task-irrelevant stimulus *duration* is bound to the *response* but not to the stimulus feature *pitch*; we observed a Duration × Response (S-R) interaction but no significant Duration × Pitch (S-S) interaction. A complete repetition or shift of the features *duration* and *response* led to a better performance compared with a partial repetition/shift of the features. Note that the data pattern can barely be explained by a potential grouping of the two low versus two high tones because we found a significant RT difference of nearly 100 ms for feature shifts between low and very low, on one hand, and between high and very high, on the other hand (we will come back to this alternative explanation in section “General discussion”).

The conclusion that the duration of stimuli is in general not bound to other stimulus features, however, seems premature. We, therefore, decided to conceptually replicate Experiment 1 by replacing the sine tones with more meaningful sounds of musical instruments to create a context of music. The reasoning is that in the domain of music, duration is a critical and informative feature because the duration of single sounds is the essence of rhythm. Even though *duration* remains task-irrelevant as a stimulus feature in Experiment 2, the stimuli used become more relevant to everyday life than the stimuli in Experiment 1 (pure sine tones as artificial tones).

## Experiment 2

Experiment 2 was an exact replication of Experiment 1, except that the four sine tones were replaced by four different musical instruments. If the (potential) relevance of duration for a given stimulus set modulates its binding with other stimulus features, then we should observe binding effects between duration and response, as well as this time between duration and the stimulus.

### Material and methods

#### Participants

Thirty students (age *M* = 24.5 years, *SD* = 9.31; range = 18–58 years; 23 self-identified as female, seven as male; two left-handed [self-report]) from the University of Regensburg participated for course credit. They all gave written informed consent prior to the experiment in accordance with the ethical standards of the National Research Committee and with the 1964 Helsinki Declaration and its later amendments. All participants reported no hearing impairment.

#### Stimuli and procedure

**Figure 5.**
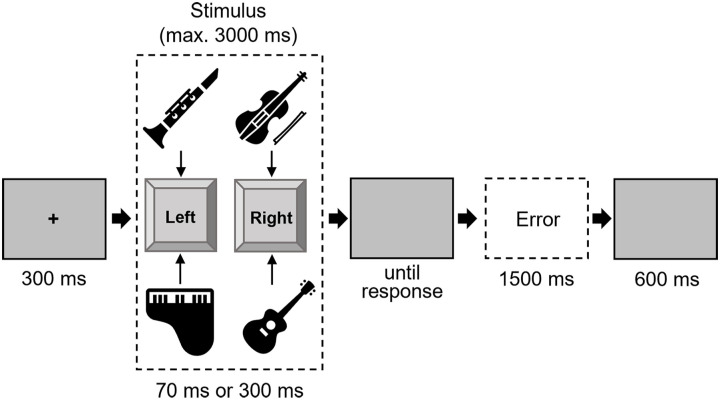
Trial procedure in Experiment 2. *Note.* The assignment of the instruments to response keys was balanced across participants. Stimuli are not drawn to scale.

The procedure of Experiment 2 mirrored that of Experiment 1, with the following modifications: the stimuli consisted of four instrument sounds (violin, clarinet, guitar, piano; all at pitch C4) with two durations (70 ms and 300 ms^6^), resulting in a total of eight stimuli. Participants were instructed to respond to two instruments with the left key and the other two with the right key. The assignment of instruments to response keys was balanced across all participants. In case of an error (participant pressed the wrong key), the word “error” appeared. If there was no response for more than 3,000 ms, the error feedback “too slow” appeared (both: white, 18 pt, Arial, bold; see Figure 5). The stimuli were created with the program GarageBand (Apple, 2018) and cut to the required length with the program Audacity (Audacity Team, 2021).

### Design and planned statistical analyses

We used the same design as in Experiment 1 but replaced the two low and two high sine tones with two different musical instruments each. Thus, our design again included three within-subject factors, each with two levels: Instrument (repetition vs shift), Duration (repetition vs shift), and Response (repetition vs shift). Any instrument shift, regardless of key assignment, was coded as an instrument shift (e.g., instrument 1—instrument 2 = shift and instrument 1—instrument 3 = shift). As a repetition of the response-relevant feature necessarily had to be answered with the same response (response repetition), the combination “instrument repetition and response shift” was not present (see Table 1 for an overview of possible condition combinations). Therefore, instead of an overall three-factorial design, we used separate two-factorial designs to investigate the respective Feature × Feature and Feature × Response interactions.

For investigating S-S binding, we conducted a 2 (*Instrument*: repetition vs. shift) × 2 (*Duration*: repetition vs. shift) ANOVA with repeated measures on both factors for *response repetition trials only*. For investigating S-R binding, we conducted a 2 (*Response*: repetition vs. shift) × 2 (*Duration*: repetition vs. shift) ANOVA with repeated measures on both factors for *instrument shift trials only*. Based on the results from Experiment 1, we expected a Response × Duration interaction (S-R binding) and, thus, partial repetition costs. The presence versus absence of the Instrument × Duration interaction (S-S binding) will be informative as to whether the potential relevance of duration for musical context has an impact. All further analyses mirror those of Experiment 1.

All aforementioned analyses were planned and determined before the experiment, i.e., a priori. Raw data files associated with this article can be found online (https://doi.org/10.5283/epub.58056).

### Results and discussion

Preprocessing was exactly the same as in Experiment 1, with one exception for extreme reaction times due to the time limit to respond. Error trials (7.57%), trials following an error trial (8.92%), trials with extreme RTs < 100 ms or > 3,000 ms (0.01%), and trials with RTs deviating more than three *SDs* from the individual condition mean (1.50%) were excluded from the RT analysis (Bush et al., 1993). Data were analysed using SPSS statistical software (IBM Corp, 2017). Bayesian analyses were conducted with the program JASP (JASP Team, 2022). Bayes factors were categorised following the approach of Wagenmakers et al. (2011) and Wetzels et al. (2011).

#### S-S binding

##### RT data

**Figure 6.**
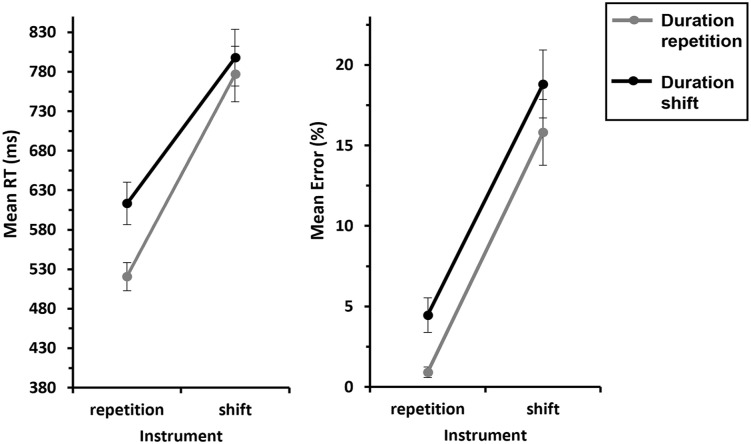
Mean RTs and errors of Experiment 2 as a function of Instrument × Duration—S-S binding. *Note.* Mean RTs (left panel) and mean error rates (right panel) as a function of Instrument (repetition vs. shift) and Duration (repetition vs. shift). Note that there are no response shifts for instrument repetitions, so we only included response repetitions in this analysis. The grey lines represent duration repetitions, and the black lines represent duration shifts. Error bars provide standard errors.

We conducted a 2 (*Instrument*: repetition vs. shift) × 2 (*Duration*: repetition vs. shift) ANOVA with repeated measures on both factors, for *response repetition trials*. This revealed a significant main effect of Instrument, *F*(1, 29) = 145, *p* < .001, $\eta_{p}^{2}$ = .833, indicating faster responses when an instrument was repeated compared with when it shifted (567 ms vs. 788 ms). The main effect of Duration also reached significance, *F*(1, 29) = 37.0, *p* < .001, $\eta_{p}^{2}$ = .561, indicating faster responses when duration was repeated compared with when it shifted (649 ms vs. 706 ms). Most importantly, the interaction of interest, Instrument × Duration, was also significant, *F*(1, 29) = 19.0, *p* < .001, $\eta_{p}^{2}$ = .396, indicating partial repetition costs: a complete repetition of the feature instrument and duration resulted in better performance compared with an instrument repetition combined with a duration shift (521 ms vs. 613 ms; see Figure 6, left panel). The inclusion Bayes factor (BF_incl_ = 6.47) for this interaction indicates that the data are 6.47 times more likely under the alternative hypothesis (interaction) than under the null hypothesis (no interaction), suggesting substantial evidence for the H_1_.

##### Error rates

An analogous ANOVA for errors yielded a significant main effect of Instrument, *F*(1, 29) = 75.8, *p* < .001, $\eta_{p}^{2}$ = .723, indicating that overall, participants made more errors when an instrument shifted than when it repeated (17.3% vs. 2.69%). Likewise, the main effect for Duration was also significant, *F*(1, 29) = 17.3, *p* < .001, $\eta_{p}^{2}$ = .374, indicating more errors for duration shifts than for duration repetitions (11.6% vs. 8.36%). The interaction Instrument × Duration did not reach significance (*F* = 0.216, *p* = .645, $\eta_{p}^{2}$ = .007; see Figure 6, right panel). The inclusion Bayes factor (BF_incl_ = 0.347) for this interaction indicates that the data are 0.347 times more likely under the alternative hypothesis (interaction) than under the null hypothesis (no interaction), suggesting anecdotal evidence for the H_0_.

#### S-R binding

##### RT data

**Figure 7.**
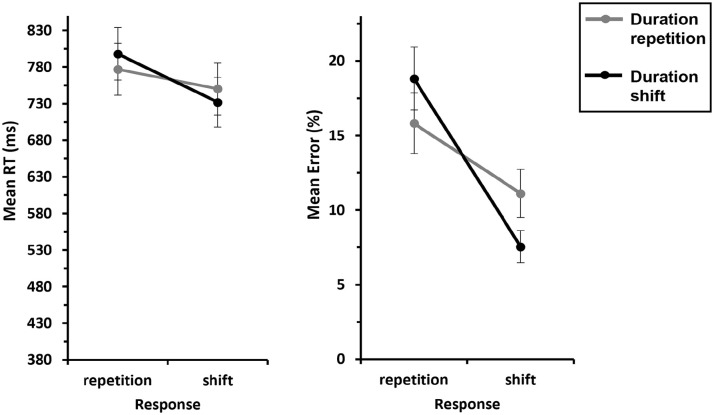
Mean RTs and errors of Experiment 2 as a function of Response × Duration—S-R binding. *Note.* Mean RTs (left panel) and mean error rates (right panel) as a function of Response (repetition vs. shift) and Duration (repetition vs. shift). Note that there are no instrument repetitions for response shifts, so we only included instrument shifts in this analysis. The grey lines represent duration repetitions, and black lines represent the duration shifts. Error bars provide standard errors.

We conducted a 2 (*Response*: repetition vs. shift) × 2 (*Duration*: repetition vs. shift) ANOVA with repeated measures on both factors for *instrument shift trials*. This revealed a significant main effect of Response, *F*(1, 29) = 46.6, *p* < .001, $\eta_{p}^{2}$ = .616, indicating faster responses for response shifts compared with response repetitions (741 ms vs. 788 ms). The interaction of interest, Response × Duration, also reached significance, *F*(1, 29) = 5.89, *p* = .022, $\eta_{p}^{2}$ = .169, indicating partial repetition costs: responses were faster when the response and duration were repeated compared with when response repeated and duration shifted (777 ms vs. 798 ms). A complete shift of response and duration led to faster responses compared with a response shift and a duration repetition (732 ms vs. 750 ms; see Figure 7, left panel). The inclusion Bayes factor (BF_incl_ = 5.11) for this interaction confirms this, indicating that the data are 5.11 times more likely under the alternative hypothesis (interaction) than under the null hypothesis (no interaction), suggesting substantial evidence for the H_1_. The factor Duration was not significant (*F* = 0.041, *p* = .842, $\eta_{p}^{2}$ = .001).

##### Error rates

An analogous ANOVA for errors yielded a significant main effect of Response, *F*(1, 29) = 36.8, *p* < .001, $\eta_{p}^{2}$ = .559, indicating that overall, participants made more errors when the response repeated than when it shifted (17.3% vs. 9.33%). The interaction of interest, Response × Duration, also reached significance, *F*(1, 29) = 21.2, *p* < .001, $\eta_{p}^{2}$ = .422, indicating partial repetition costs: responses were less error-prone when the response and duration were repeated compared with when response repeated and duration shifted (15.8% vs. 18.8%). A complete shift of response and duration resulted in fewer errors compared with shifting the response and repeating the duration (7.54% vs. 11.1%; see Figure 7, right panel). The inclusion Bayes factor (BF_incl_ = 46.6) for this interaction confirms this, indicating that the data are 46.6 times more likely under the alternative hypothesis (interaction) than under the null hypothesis (no interaction), suggesting very strong evidence for the H_1_. The factor Duration was not significant (*F* = 0.317, *p* = .578, $\eta_{p}^{2}$ = .011).

#### Binding effect

**Figure 8.**
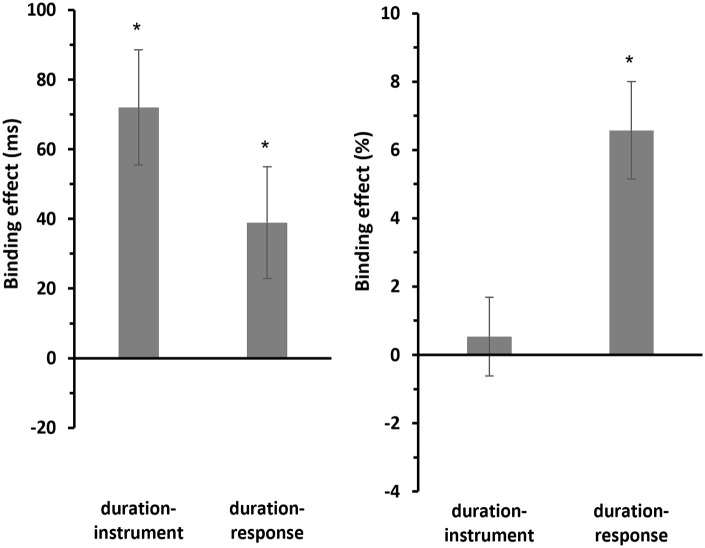
Mean binding effects for RTs and errors of Experiment 2. *Note.* Mean binding effects for RTs (left panel) and errors (right panel). For calculation of the binding effect, see text. Error bars provide standard errors.

Post hoc one-sample *t*-tests (two-sided) revealed that the binding effect for duration-instrument binding (*M* = 72.0 ms, *SE* = 16.5 ms) was significantly different from zero, *t*(29) = 4.36, *p* < .001, *d* = 0.796, 95% CI for Cohen’s *d* [0.379, 1.203], and BF_10_ = 181. Similarly, the binding effect for duration-response binding (*M* = 39.0 ms, *SE* = 16.0 ms) was also significantly different from zero, *t*(29) = 2.43, *p* = .022, *d* = 0.443, 95% CI for Cohen’s *d* [0.064, 0.815], and BF_10_ = 2.36 (see Figure 8, left panel).

For the error rates (see Figure 8, right panel), the post hoc one-sample *t*-tests (two-sided) showed that the binding effect for duration-instrument binding (*M* = 0.536%, *SE* = 1.15%) was not significantly different from zero, *t*(29) = 0.465, *p* = .645, *d* = 0.085, 95% CI for Cohen’s *d* [−0.274, 0.443], and BF_10_ = 0.215. However, the binding effect for duration-response binding (*M* = 6.58%, *SE* = 1.43%) was significantly different from zero, *t*(29) = 4.60, *p* < .001, *d* = 0.840, 95% CI for Cohen’s *d* [0.417, 1.252], and BF_10_ = 328.

#### Post hoc analysis

Given the discrepant results concerning the S-S interaction of the two experiments, the question arises whether the differences are true or due to a lack of power to detect a smaller effect in Experiment 1. Therefore, we conducted an additional ANOVA with *Experiment* (1 vs. 2) as a between-subjects factor and *Relevant Stimulus* (repetition vs. shift) and *Duration* (repetition vs. shift) as within-subject factors (again, using *response repetition trials* only; see Table 1) to gain more clarity about the possible existence of a difference in the results of the S-S analyses of both experiments.^7^ The higher-order interaction Relevant Stimulus × Duration × Experiment was highly significant, *F*(1, 58) = 17.1, *p* < .001, $\eta_{p}^{2}$ = .228, confirming the significant difference between Experiments 1 and 2 in RT data with regard to S-S binding.

The results of Experiment 2 partially confirmed and extended the findings from Experiment 1. As observed in Experiment 1, partial repetition costs for duration-response binding occurred in both RTs and error rates. In contrast to Experiment 1, the Instrument × Duration interaction was also significant, indicating S-S binding. Together with the post hoc analysis, comparing S-S binding between experiments, this suggests that the potential relevance of duration for a given stimulus set (here, sine tones vs. musical instruments) has an effect on binding.

## General discussion

This study aimed to determine whether stimulus duration is bound to another stimulus feature, to the response, or to both. The results suggest robust S-R binding of duration and response, whereas S-S binding of duration to another stimulus feature (indicated by an S-S interaction) was only found in Experiment 2 with four different musical instruments but not in Experiment 1 with four different sine tones. More precisely, in Experiment 1, we used four different sine tones as the stimulus set, resulting in a Duration × Response interaction, indicating partial repetition costs and, thus, duration-response binding (S-R). The Pitch × Duration interaction, on the contrary, did not reach significance and was associated with a negligible effect size estimate ( $\eta_{p}^{2}$ < .001), indicating a lack of S-S binding in Experiment 1. In Experiment 2, the stimulus set was replaced with sounds from musical instruments to create a context where duration is potentially relevant, although the stimulus duration itself remained task-irrelevant. The results again showed S-R binding (confirming the results of Experiment 1) and, this time, also a significant interaction between instrument and duration with a large effect size ( $\eta_{p}^{2}$ = .396), indicating partial repetition costs and, thus, S-S binding. Post hoc analyses comparing both experiments confirmed these results. This is particularly important because the absence of an S-S effect in Experiment 1 and the presence of an S-S effect in Experiment 2 alone are not sufficient to draw a clear conclusion about the difference between the two experiments regarding this effect. However, the significant higher-order interaction between Experiment, Relevant Stimulus, and Duration observed in the post hoc analysis strengthens the conclusion that the two experiments differ significantly with regard to the S-S effect.

The robust results of S-R binding in both experiments are consistent with previous findings in the S-R binding literature, which have repeatedly shown that stimulus features, such as colour, shape, location, size, word identity, and sine tones are bound to the response (e.g., Hommel, 2007; Hommel & Colzato, 2004; Horner & Henson, 2009, 2011; Moeller et al., 2015; Moeller & Frings, 2019a; Mordkoff & Halterman, 2008; Rothermund et al., 2005; Schöpper & Frings, 2022; Zehetleitner et al., 2012). Previous findings demonstrating the temporal integration of duration into auditory event files (see Bogon, Thomaschke, & Dreisbach, 2017) left open the question of whether duration is bound to the stimulus (S-S) or to the response (S-R). In the study of Bogon, Thomaschke, and Dreisbach (2017), each repetition/shift of the task-relevant stimulus feature (Experiment 1: pitch; Experiment 2: loudness) also involved a repetition/shift of the response, making it difficult to conclusively determine the binding of duration. The results of this study (S-R binding in both experiments) indicate that duration is reliably bound to the response, at least within auditory files. Considering that in the experiments by Bogon, Thomaschke, and Dreisbach (2017), as well as in Experiment 1 of this study (S-R binding and no clear evidence for S-S binding), the stimulus set consisted of pure sine tones, it is reasonable to assume that the integration of stimulus duration occurred due to S-R binding.

One might argue that the lack of a significant interaction between pitch and duration (S-S binding) in Experiment 1 was due to grouping. Specifically, the two low tones and the two high tones could have been grouped into overarching categories of low versus high. This could perfectly explain the lack of S-S binding (see, e.g., Bogon, Eisenbarth, et al., 2017; Dreisbach & Haider, 2008; no binding for task-rule mapping). However, responses at pitch shifts (between low and very low and between high and very high) were significantly slower (by almost 100 ms) than pitch repetitions. This is hard to reconcile with the view that low and very low (and high and very high) pitches were grouped into low- versus high-pitch tones. However, we admit that grouping cannot entirely be ruled out and might have contributed to preventing a binding effect.

However, we propose an alternative explanation, namely that the binding of duration depends on the specific context (artificial vs. musical). This means that the integration of the stimulus duration to another stimulus feature depends on the type of stimulus set. More specifically, the results of Experiment 2 suggest a potential role of the relevance and informational value of the duration for that stimulus set, even if the duration itself is not task-relevant. For a stimulus set of pure sine tones (Experiment 1: no S-S binding), the stimulus duration contains no potentially relevant information, but for a stimulus set of musical instrument sounds (Experiment 2: S-S binding), it does. This is because, in the domain of music, the duration of individual sounds constitutes the essence of rhythm (Hauser & McDermott, 2003; Honing, 2013). One could argue that in Experiment 1, individual tones of different durations were already presented and, thus, the duration should already have had relevance for the stimulus set. However, it is essential that the presentation of such single artificial sine tones does not create a context of music. In contrast, the sounds of real musical instruments do create a musical context and, thus, a stimulus set for which duration is potentially relevant and informative. In Experiment 1, such a context was not present and, thus, no S-S binding emerged or was not strong enough to be detected in the results. This would mean that the binding of duration as a stimulus feature to another stimulus feature, at least in the auditory context, depends on the potential relevance of duration to the stimulus set and, thus, the indirect task-relevance attributed by the individual. At first glance, our findings seem to correspond to Colzato et al. (2006), who showed that bindings between colour and object are stronger for naturally occurring feature combinations like yellow banana and red strawberry. However, and different from their approach, we did not use musical sounds that have long-learned associations with either long or short durations. Instead, we suggest that some feature categories might more easily bind to each other than others (like durations to musical sounds as opposed to durations to single artificial sine tones).

To sum up, we found robust S-R binding between duration and response in both experiments, independent of the stimulus set. The findings regarding S-S binding are not yet clearly interpretable but suggest that binding between different stimulus features can be modulated by the potential relatedness of the features involved. This is indicated by the lack of S-S binding in Experiment 1 (where duration has no informational value for artificial sine tones) and the occurrence of S-S binding in Experiment 2 with musical sounds, for which duration is always informative. This shows once again that context plays a role in binding processes, not only as a possible feature that can be integrated (e.g., Benini et al., 2023; Frings & Rothermund, 2017) but also as a moderator that modulates S-S binding. Either way, the results of this study provide new impetus to investigate binding mechanisms in the musical context in more detail.

## Notes

1. Due to the large number of available articles, we cite only one paper as an example that showed the effect for the first time.

2. Note that due to the design, the two factor combinations “pitch repetition—duration repetition—response shift” and “pitch repetition—duration shift—response shift” necessarily cannot exist.

3. PRDS = pitch repetition and duration shift; PRDR = pitch repetition and duration repetition; PSDS = pitch shift and duration shift; PSDR = pitch shift and duration repetition. For *response repetition trials only.*

4. RRDS = response repetition and duration shift; RRDR = response repetition and duration repetition; RSDS = response shift and duration shift; RSDR = response shift and duration repetition. For *pitch shift trials only.*

5. Preprocessing based on Köllnberger et al. (2023); Kunde et al. (2002); Kunde et al. (2004).

6. The presentation durations for short and long are longer than in Experiment 1, as otherwise the identification of the instrument sounds would not have been possible or would have been too difficult for the short duration.

7. We would like to thank the editor and an anonymous reviewer for suggesting this analysis.
